# The performance of integrated health care networks in continuity of care: a qualitative multiple case study of COPD patients

**DOI:** 10.5334/ijic.1527

**Published:** 2015-07-20

**Authors:** Sina Waibel, Ingrid Vargas, Marta-Beatriz Aller, Renata Gusmão, Diana Henao, M. Luisa Vázquez

**Affiliations:** Health Policy and Health Services Research Group, Health Policy Research Unit, Consortium for Health Care and Social Services of Catalonia, Barcelona, Spain; Department for Paediatrics, Obstetrics and Gynaecology, Preventive Medicine, Universitat Autònoma de Barcelona, Bellaterra, Spain; Health Policy and Health Services Research Group, Health Policy Research Unit, Consortium for Health Care and Social Services of Catalonia, Barcelona, Spain; Health Policy and Health Services Research Group, Health Policy Research Unit, Consortium for Health Care and Social Services of Catalonia, Barcelona, Spain; Health Policy and Health Services Research Group, Health Policy Research Unit, Consortium for Health Care and Social Services of Catalonia, Barcelona, Spain; Health Policy and Health Services Research Group, Health Policy Research Unit, Consortium for Health Care and Social Services of Catalonia, Barcelona, Spain; Health Policy and Health Services Research Group, Health Policy Research Unit, Consortium for Health Care and Social Services of Catalonia, Barcelona, Spain

**Keywords:** continuity of patient care, integrated delivery systems, qualitative research, chronic obstructive pulmonary disease, patient–physician relationship, clinical management continuity, clinical information continuity

## Abstract

**Background:**

Integrated health care networks (IHN) are promoted in numerous countries as a response to fragmented care delivery by providing a coordinated continuum of services to a defined population. However, evidence on their effectiveness and outcome is scarce, particularly considering continuity across levels of care; that is the patient's experience of connected and coherent care received from professionals of the different care levels over time. The objective was to analyse the chronic obstructive pulmonary disease (COPD) patients’ perceptions of continuity of clinical management and information across care levels and continuity of relation in IHN of the public health care system of Catalonia.

**Methods:**

A qualitative multiple case study was conducted, where the cases are COPD patients. A theoretical sample was selected in two stages: (1) study contexts: IHN and (2) study cases consisting of COPD patients. Data were collected by means of individual, semi-structured interviews to the patients, their general practitioners and pulmonologists and review of records. A thematic content analysis segmented by IHN and cases with a triangulation of sources and analysists was carried out.

**Results:**

COPD patients of all networks perceived that continuity of clinical management was existent due to clear distribution of roles for COPD care across levels, rapid access to care during exacerbations and referrals to secondary care when needed; nevertheless, patients of some networks highlighted too long waiting times to non-urgent secondary care. Physicians generally agreed with patients, however, also indicated unclear distribution of roles, some inadequate referrals and long waiting times to primary care in some networks. Concerning continuity of information, patients across networks considered that their clinical information was transferred across levels via computer and that physicians also used informal communication mechanisms (e-mail, telephone); whereas physicians highlighted numerous problems of the information system, thus the need to use informal communication channels. Finally, regarding continuity of relation, patients of some networks pointed out high turnover of personnel - being frequently seen by locum doctors or assigned to new physicians - which hindered the development of a trusting relationship.

**Conclusion:**

Study findings suggest a generally perceived adequate performance of IHN in continuity of care but also the existence of a series of difficulties related to all continuity types. Results can provide opportunities to improving the care process of COPD patients but also of patients with other conditions who receive care across the primary and secondary care level.

## Introduction

Health care fragmentation, which is health care that lacks coordination among the involved providers, is considered to lead to worse clinical outcomes because of poor quality of care due to duplications of diagnostic tests, perilous polypharmacy and conflicting care plans [1,2]. This is considered to be particularly relevant to patients with chronic conditions or multi-pathologies who tend to receive care from different professionals in various settings and institutions, thus, are more likely to be exposed to fragmented health care delivery [3]. Among the responses to fragmentation, health ministries of different countries and multilateral organisations are promoting the development of integrated health care networks (IHN) [4] (also called integrated health services delivery networks [5] or integrated delivery networks [6]), which are defined as networks of organisations that provide or arrange to provide a coordinated continuum of health services to a defined population and are willing to be held clinically and fiscally accountable for the outcomes and health status of the population [7]. Their ultimate objective is to improve equity of access, quality of care and global system efficiency, by means of enhancing access, coordination and continuity of care [8]. IHN are characterised by their integration width (number of different services provided across the care continuum), integration depth (extent to which a given service is provided at multiple operating units within the network), geographic concentration of services, level of internal production of services and their inter-organisational relationship [7].

IHN were first promoted in the United States to address the problems of its highly fragmented health care system [9], and in countries with health systems based on competitive insurance markets (managed competition model). Since the early 1990s, this kind of initiative emerged in countries with a national health system, where health care management was devolved to a lower tier of government, such as in Spain [10,11]. The Spanish National Health System is financed by taxes, and at the time of the study, offered universal coverage and free access at the point of delivery; and is decentralised into regional health services [12]. Health care provision is organised into levels of complexity, where primary care is the gatekeeper and responsible for coordinating the patient's care along the care continuum [13] and secondary or specialised care acts as a consultant of primary care and is responsible for more complex care [12,14]. In the autonomous community of Catalonia, the health care system is characterised by a split of the financing and provision functions. The provision of services is the responsibility of a number of contracted providers: on the one hand, a public company, the Catalan Health Institute (Institut Català de la Salut), on the other, consortia, municipal foundations and private foundations (mainly non-profit but also for profit) [15]. This diversity of providers, which involves a greater risk of care fragmentation, has led to different arrangements for the joint management of primary, secondary and long-term care, as for example the creation of IHN. At the moment, twenty provider networks with different degrees of care integration can be identified [10,11].

According to Vázquez et al. [16], the performance of IHN should be analysed with regard to their final outcomes (equity of access, efficiency and quality of care) but also their intermediate outcomes (continuity of care, care coordination and access) and taking into account the internal processes developed by the networks to achieve their objectives (e.g., the organisational structure or the organisation of the care provision) and the macro- and micro-level contexts in which the networks perform [16]. Continuity of care, the focus of the present article, is defined as one patient experiencing care over time as connected and coherent with his or her health needs and personal circumstances [17]. Three types can be distinguished: *continuity of clinical management*, that is the patient's perception of the receipt of the different services in a coherent way responsive to his or her changing needs; *continuity of information*, that is the patient's perception of the availability and use of information on past events and personal circumstances; and *relational continuity*, that is the patient's perception of an ongoing, therapeutic relationship with one or more provider(s) [17,18]. Whereas continuity of clinical management and information refer to the patient's perception of the interaction among providers and can be analysed across levels of care; relational continuity can only be analysed in each care level separately [18].

Continuity of care has been garnering more attention in the last years, accompanied by an increased number of publications that aimed to improve the conceptual framework [19,20] and to identify conceptual boundaries to related concepts, such as coordination, integration or patient-centred care [21], as well as to describe patients’ experiences and perceptions of continuity of care employing qualitative methods, with a predominance of the selection of primary care settings [22] of national health systems, mainly the United Kingdom but also Canada [22,23]. Existent studies on patients’ perceptions further concentrate on analysing relational continuity, that is, one level of care; whereas continuity of clinical management and information across levels of care have been studied to a much lesser degree [22]. Furthermore, studies focus on chronic conditions, especially diabetes [22,23] and cancer [23], and mentally ill persons [22,23]. The perception of patients with chronic obstructive pulmonary disease (COPD) of continuity of care was analysed by means of qualitative methods in a few studies conducted in Denmark [24], Great Britain [25] and Canada [26], that revealed a number of limitations of all continuity types throughout the care process, such as limited access to services [24,25], lack of proactive primary or secondary follow-up after hospitalisation [25] or ineffective communication between physicians [24,26].

Factors which could, according to patients, influence i.e. facilitate or distract from the three continuity of care types only emerged as a by-product of qualitative study results, such as the shortage of health professionals or lack of time [27,28]. In quantitative research, the analysis focuses on finding associations between the perception of continuity and individual factors, such as sex, age, educational level [29,30] or morbidity [29,31,32]; however, organisational factors which could, according to patients, influence continuity of care were scarcely studied [33,34]. The need for an in-depth analysis to understand the full complexity of the phenomenon has been postulated [29].

Despite the high expectations that have been attached to IHN [35] empirical evidence on the effectiveness and outcome is scant [5,16,35], and it has been questioned that they can actually ensure continuity of care [36,37]. Limited available results on continuity of clinical management and information in IHN in Canada [38] and the United States [39] based on cross-sectional surveys are contradictory; showing, on the one hand, that the patient experience of continuity is positively associated with the existence of formal and informal inter-organisational arrangements between various providers [38]; and on the other hand, limited associations [39].

IHN of Catalonia were the subject of different studies, mainly for their characterisation and analysis of care coordination [11,40,41], however, concerning their performance in continuity of care, studies are very limited. Results from a patient survey suggest that patients attended to in organisations in which primary and secondary care services were managed by the same entity experienced better continuity of care, and that the most favourable reports were given by patients who were attended to at the services managed by a single entity under public law [29].

This article contributes to the analysis of IHN performance by analysing the COPD patients’ perceptions of continuity of clinical management and information across care levels and continuity of relation in IHN of the public health care system of Catalonia.

## Methods

### Study design

An interpretative, qualitative study was conducted using a phenomenological approach and a multiple case study design. The theory of phenomenology focuses on exploring how individuals make sense of the world and aims to provide insightful accounts into their subjective experiences [42]. The purpose of case studies is to gather comprehensive, systematic and in-depth information about each case of interest [43]. Case studies do not aim to generalise the results from a statistically representative sample, rather to generate theory that stem from the specificities of concrete cases [44]. By adopting a case study design, the phenomenon of continuity of care can be understood in its multifaceted manner using different sets of information [45].

### Study sample

A theoretical sample [46] was designed consisting of two stages. In the first stage, four IHN of the Catalonian public healthcare system were selected applying the following criteria: (1) provision of primary and secondary care (comprising at least one hospital), (2) joint management for over 5 years and (3) delivery of health care services to a defined population. The selected networks showed similarities regarding the level of internal production of services (100%) but differences regarding the integration depth (contracted centres) and inter-organisational relationship (type of agreement). The networks introduced different types of care coordination mechanisms in general and for COPD specifically; ranging from an implemented single mechanism (shared electronic medical records, shared clinical guidelines, COPD patient registers, etc.) to a combination of mechanisms in a comprehensive programme (disease or case management) (Table 1) [10,11]. In the second stage, two study cases of each IHN were selected; a study case consisted of one COPD patient (Table 2). COPD was selected as the tracer condition since patients require care provided by primary and secondary care professionals over time [47,48]. Patients were selected according to the following criteria: (1) confirmed COPD diagnosis, (2) chronicity ≥ 5 years, (3) having received care for at least 2 years at the network and (4) utilisation of both levels of care in the last 6 months. For discursive variation, the distance from the primary care centre to the hospital was taken into consideration during the selection process. An equal distribution of female and male patients was targeted; however, only one female patient who met the predefined criteria accepted to participate.

### Data collection

Data were retrieved by two trained researchers (SW and RG) by means of individual, semi-structured interviews with COPD patients, his/her general practitioner (GP), his/her pulmonologist or case manager (secondary care nurse), that the patients identified as the most relevant health professional in their COPD care, and the review of patients' medical records (Table 2). Specific topic guides were elaborated for patients (Annexe 1) and health professionals (Annexe 2) comprising two parts: one to reconstruct the clinical trajectory and one to explore their perceptions of the three types of continuity of care (clinical management, information and relation) following Reid et al.'s [18] conceptual framework. The topic guide was used as a prompt to the interviewers to ensure that relevant issues on continuity of care were covered considering the open-ended nature of the interview. Patients were selected by the researchers from a list provided by the organisation and then contacted by telephone by a secretary of each organisation. When agreed to participate, interviews were performed depending on the patients’ preferences, in most cases at their homes but also at a café or in a quiet room at the primary care centre. In almost all cases, two interviews with each patient were necessary to attain data saturation, i.e. an additional interview would not provide further information. Family carers of two patients participated in the interview, providing relevant data, which were included in the analysis. The interviews with health professionals (GP and pulmonologist or case manager) were conducted in the health care facilities. Field notes on preliminary ideas and reflections were taken during and after the interviews to enhance reflexivity, which refers to the recognition of influence a researcher brings to the research process [46]. Interviews ranged from 30 to 80 minutes in length, were audio-recorded, transcribed verbatim and anonymised. The patients’ medical records were reviewed using a structured guide (Annexe 3) to contrast the data obtained from the interviews.

### Data analysis

A thematic content analysis was conducted with support of the software Atlas-ti 5.0. Data were segmented by case and information source. The discovery and pre-analysis phase consisted of the iterative reading of the interviews, followed by a mixed generation of categories [49], i.e. categories derived from the conceptual framework reflected in the topic guides and emerged from the data. Transcripts were coded, categories developed and refined as new sections of text were examined. Common patterns were identified by looking at regularities, convergences and divergences in data through a process of constant comparison, going back and forth between data. A study narrative was written for each case that represented a comprehensive, descriptive data presentation (case-oriented analysis including the COPD patient, his/her health professionals and medical records). In a second step, a category-oriented analysis across IHN was conducted to identify key categories [50] of the perception of continuity of care.

### Quality control and ethical considerations

Quality of research results was guaranteed by the triangulation of information derived from different sources, which ensured comprehensiveness of data collection and facilitated a more reflective data analysis [51], and by the triangulation of analysis by three researchers who were knowledgeable about qualitative research and the phenomenon of continuity of care, so as to enhance accuracy of findings. With regard to ethical considerations, written informed consent was sought from each participant and data were masked to ensure anonymity and confidentiality.

## Results

The analysis of the cases presents the COPD patients’ perceptions of both continuity and discontinuity that depended on the continuity type (clinical management, information and relation) and, in some concrete aspects, on the IHN where they were attended to. Health professionals’ opinions and reviews of medical records generally showed congruencies with patients' opinions, and only in a few aspects, differing results.

### Continuity of clinical management

Patients across IHN identified their specialist to be responsible for their COPD care and highlighted that follow-up visits were programmed adequately, GPs followed instructions received from the other care level, referrals were done when necessary and access to urgent care was appropriate; however, patients also indicated too long waiting times to secondary care in non-urgent cases.

#### Clear distribution of COPD care responsibilities

COPD patients across IHN considered that responsibilities for their COPD treatment were clearly distributed across care levels: GPs were in charge of resolving minor health problems, prescribing the medication indicated by pulmonologists: *(The prescription) is done together with the specialist because what the specialist says the GP prescribes (Patient of IHN 1)*, and in some networks, of treating exacerbations; pulmonologists, in turn, were considered to be responsible for the overall care of their condition including diagnosis, decision-making on treatment possibilities and performing tests due to their technical knowledge and competence: *Well, the responsible person for treating my disease, sure, apart from the GP, I believe it's the specialist; it's he who knows more or less (…) Sure, the specialist is responsible; the one who makes the diagnosis is the specialist (Patient of IHN 4)*. Physicians of most IHN agreed on clear roles regarding regular monitoring of COPD care, with the responsibility either being assumed by the GP or the pulmonologist depending on the severity of the conditions: *If it is a mild or moderate COPD, basically, in this case, I assume the responsibility, and when I have any questions or problems regarding the patient, then I refer him to secondary care. Generally, severe COPD is monitored by the hospital specialists (GP of IHN 1)*. Opinions of physicians of IHN 3, however, differed regarding which care level was finally in charge of monitoring the patient: *Well, this patient, I am not sure if we should control him because it's not a patient with numerous hospitalizations (…) I am not certain to what extent his GP should control him (Pulmonologist of IHN 2). His day-to-day control visits are done here (in primary care) because that's the main access he has to the health system (GP of IHN 2)*. Furthermore, physicians of IHN 1 and IHN 4 disagreed on which care level was responsible for providing acute care (treating exacerbations) and follow-up visits after hospitalisation and emergency care visit: *(After a emergency care visit), the patient is usually referred to me (Pulmonologist of IHN 4); (…) he sees his GP for follow-up, to move forward after having had an episode (GP of IHN 4)*. According to the review of the clinical histories, physicians of both care levels of IHN 3 and IHN 4 ordered medical tests; thus, no responsible person was identified.

Patients generally considered that follow-up visits were programmed adequately by both primary and secondary doctors with or without regularity, and within an adequate time span depending on the severity of the illness: *The distance between the visits depends on how I feel (Patient of IHN 4)*. Physicians of both care levels and clinical histories concurred that no regularity of visits was established and the patient was attended to when necessary: *The visits with COPD patients are programmed according to his needs and considering his exacerbations (GP of IHN 3)*.

#### Coherence of health care across the different levels

Patients across IHN perceived that GPs followed the instructions received from the specialists and incorporated the treatment into their medication plan, which was confirmed by the physicians. Nevertheless, patients of IHN 2 indicated that pulmonologists disregarded recommendations from the other care level: *They all work on their own. If you go upstairs (to the pulmonary department) and say, ‘because my doctor said to me that…’ They respond to me, ‘here, your doctor doesn't say anything, here, I am the physician’, and I stop talking (Patient of IHN 2)*.

In all IHN, patients perceived that they were referred to secondary care when necessary and that primary care follow-up visits were programmed after receiving secondary care when needed, i.e. due to a change of treatment by the pulmonologist, reconciliation of medication and discharge from secondary outpatient care because the condition had stabilised. Only in one occasion the referral was considered to be inadequate since the pulmonologist did not solve the heath problem: *Once she (the pulmonologist) said to me that I should not come back! She said that I must not come to her anymore when I have this problem; (that) I have to go and see the GP instead (Patient of IHN 4)*. In agreement with patients, physicians of both care levels perceived that referrals were generally appropriate; except for pulmonologists of IHN 2 who pointed out some inadequate referrals, where the health problem should have been solved in primary care; and GPs of IHN 1 who indicated that pulmonologists took over too many responsibilities: *In secondary care, there is a tendency to assume lots of tasks, but once they have taken these over, they say that they're overloaded and then they refer the patient to us (GP of IHN 1)*.

#### High accessibility to primary care and during exacerbations but long waiting times to non-urgent secondary care

During exacerbations of COPD, that is acute care, patients in all IHN considered both care levels accessible given rapid or immediate access (face to face or telephone) to their GP or pulmonologist directly or via an urgent referral by their GP to secondary care (inpatient or emergency care): *when the GP considers that I am really in a bad condition, then rapidly to the hospital (Patient of IHN 4)*. In IHN 2, the COPD case coordinator was perceived to facilitate access to the right care level within an adequate time span for urgent care. Regarding non-urgent care, patients across IHN perceived that primary care was accessible because they could schedule medical visits easily (by telephone or face-to-face), solicit as many visits as needed and were seen within a reasonable time span (a few days): *They schedule me a visit quite fast (…) maybe I wait 2 or 3 days, 4 maximum (Patient of IHN 3)*; whereas waiting times for the specialist in non-urgent cases were perceived to be inadequately long (several months): *If it's not urgent, it takes a long time; it takes months (Patient of IHN 4)*; attributed to a lack of doctors. Professionals across IHN agreed on fast access for exacerbations directly or by phone; and, except for IHN 3, long waiting times to non-urgent secondary care: *The only thing that could be better with this patient is the waiting list for the electrocardiogram; a patient with COPD and oedemas. Imagine waiting eight months, that's really annoying! (GP of IHN 3)*. In contrast to patients, GPs of IHN 2 and 3 highlighted too long waiting times for scheduling a primary care visit because of work overload. Professionals of some IHN considered that COPD patients who were frequent users usually knew which professional they had to approach to be seen more quickly because of their acquired knowledge of the system: *He is a chronic patient who knows very well how the hospital works and how we work here in the primary care centre. That is why I believe he knows how to get in touch with the person he wants to see (GP of IHN 2)*.

### Continuity of information

Patients across IHN perceived that information on their medical visits, test results and diagnosis was accessible to all health professionals via computer, and generally used by their assigned professionals however, not always by emergency doctors. Only professionals of two IHN confirmed the existence of information transfer across levels via their electronic medical records of different sorts; however, physicians across the IHN highlighted numerous problems in accessing them, and thus the need to use informal communication channels, also described by the patients.

#### Perceived transfer of clinical information via computer but limited uptake by emergency doctors

In all IHN, COPD patients perceived that medical information on their condition was transferred across care levels within the network they were attended to given that they were spared from repeating information on their diagnosis, treatment or visits in other settings and from carrying test results. Information was considered to be transferred automatically via computer, accessible to all health professionals: *Physicians have access to our information with their computers which are interconnected, those from the hospital with the whole territory to which it belongs (Patient of IHN 1)*. Patients highlighted that their medical records were usually consulted by their assigned professionals: *Nowadays everything is stored in the computers. My GP performs a medical test and writes down the results in the computer and then when I see the pulmonologist she looks it up and already knows (Patient of IHN 3)*; but only limitedly when they were attended to by emergency physicians who prioritise the rapid provision of care; thus rely on the patients’ accounts: *You arrive at the emergency room and they don't look up your history; you explain to them more or less what you have (…) They don't stop to look at the medical records (Patient of IHN 2)*. In contrast, only physicians of IHN 3 and IHN 4 generally described the information transfer by means of the implemented electronic COPD patient registers or single electronic medical record system; whereas physicians of IHN 1 and IHN 2 expressed that information transfer was limited: *We encounter problems to access some tests of them (primary care), in this case, some analytical tests (Pulmonologist of IHN 1)*. Limitations of information sharing on COPD patients, however, were expressed by physicians of all IHN, such as the difficult handling of the information system, clinical data not being registered: *If there is nothing written down, it's a disaster, although we have an excellent information system (GP of IHN 4)*, data being difficult to understand or high quantities of registered data that, together with constant time pressure, impeded the localisation of specific information. These difficulties were also confirmed by the review of the clinical histories in all IHN, which, according to physicians, would lead to duplication of medical tests and potentially harmed the patient.

#### Informal communication especially when physicians know each other

Some patients in all IHN perceived that their doctors used informal communication (via e-mail and telephone) to inform about their patient referral or to adapt their medication plans: *Depending on the problem, if it is complicated, the GP refers you to the specialists (…) and when he then wants to prescribe the medication, they talk to each other, they coordinate themselves (Patient of IHN 1)*. Physicians in all IHN coincided in the use of informal communication, especially to discuss complex issues regarding the COPD patients (curbside consultation): *Very often they (GPs) call or send me an e-mail when they have a problem. I think that's agile and communication exists, and above all it's easy to realize (Pulmonologist of IHN 4)*. Doctors considered that this type of communication compensated for the difficulties in the use of the information system and was facilitated by mutual knowledge and co-location of physicians: *Regarding COPD, the communication is not too bad: we have the specialist here (in the primary care centre), therefore, the possibility to talk to him in person (GP of IHN 3)*. Nevertheless, some GPs of IHN 1 and IHN 3 highlighted insufficient time to communicate: *The specialists are relatively accessible by two means: one via e-mail (…) and the other via a telephone consultation. However, what happens is that for both you need some space of time (…) and that's a topic that is not considered by the organization (GP of IHN 1)*; and the secondary care physician's lack of willingness to collaborate, for example, by not reporting back after a curbside consultation on the COPD patient: *It's useless that they tried to implement a single clinical history and then the specialists work on their own; I can't find that logical; because very often receiving feedback doesn't work: that I make a curbside consultation and they respond to it; only occasionally a specialist responds, only every now and then (GP of IHN 3)*.

### Continuity of relation

In all IHN, only patients who were regularly attended to by their assigned GPs and specialists over a longer period of time developed a trusting relationship, while the patients who were frequently seen by locum doctors or were assigned to a new primary or secondary care physician found it difficult to establish a bond. In addition to being attended to regularly by the same professional, the perceived technical quality, the physician's positive attitude and an effective patient–physician communication were essential factors for developing a trusting relationship.

#### Some stability but also turnover and frequent visits by locum doctors

The perception of COPD patients of being attended by the same professionals differed across networks and care level. Some patients declared being seen mainly by their assigned GP (especially IHN 2 and IHN 4) and pulmonologist (IHN 4); whereas some patients of the other networks highlighted frequent visits by locum doctors together with being assigned to new primary and secondary care physicians in various occasions: *She was our GP for years but by leaving she let us down (…) Until I don't have a permanent GP, they just give us locum doctors. They change our physicians constantly (Patient of IHN 1)* Some locum doctors did not show interest in the patient care: *She (the new GP) is not my physician and also doesn't show any interest in me. I go and see her and say ‘look, I have got a bad cold and I have…’, ‘ah, so I prescribe you antibiotics.’ But that's it. I mean, she doesn't look at you. I suppose she doesn't care as much because is neither her consultation nor I am her patient (Patient of IHN 1)*. High turnover was perceived to be caused by a shortage of personnel, and the assignation to a new pulmonologist was related to an administrative error or a deterioration of the condition: *When they change the pulmonologist this means that something is going wrong. The first thing I thought was that I wasn't doing so well anymore (Patient of IHN 2)*. In accordance with patients’ accounts, clinical histories and GPs of IHN 1 confirmed a high rotation of personnel in primary care: *Unfortunately, the zone where he belongs to is experiencing a continuous replacement of primary care professional for at least the last 3 to 4 years (GP of IHN 1)*.

#### Establishing a trusting patient–physician relationship over time

Patients of IHN 2 and IHN 4 expressed having developed a close relationship that was characterised by trust, familiarity with the care received and a sense of affiliation and loyalty, i.e. disagreement with handoff or assignation to a new physician: *From the beginning on (…) I still have the same GP. They suggested me to change the doctor when they were assigning patients to different physicians, but I asked if I could continue with the same because I was satisfied with him (Patient of IHN 4)*. From the different patient discourses emerged that a close and trusting relationship with the primary and secondary care physician was facilitated - in addition to being regularly seen by the same physician for a longer period: *You visit the GPs more frequently (than the specialists) and because of seeing them more frequently you develop trust in them (Patient of IHN 3) *- by three aspects: first, technical quality (accurate physical examinations, administration of all necessary tests, adequate and timely referrals, uptake of information from electronic medical records, sufficient consultation time) and specially with pulmonologists, their technical knowledge and competence to diagnose and treat COPD: *I trust them (the specialists). I believe that they are sufficiently competent to know what I have and how to treat it (Patient of IHN 1)*; second, an effective patient–physician communication expressed in different ways (provision of detailed and clear explanations of the disease and treatment with tactfulness, and the patient's confidence to ask: *She (the GP) is trustworthy, so you talk with her and ask her questions (…) without feeling embarrassed* (Patient of IHN 2)); and third, the physician's positive attitude (showing understanding, attentiveness, kindness, seriousness, openness, involvement and demonstrated interest in the patient's well-being and care). Patients highlighted that a long-term relationship based on trust led to feelings of security and confidence, as well as to adherence to the medication plan. Physicians of both care levels (primary care: IHN 2 and IHN 4, secondary care IHN 1) also perceived the existence of a friendly relationship with their CODP patients based on loyalty and mutual trust: *She trusts us, I think that's good, and we also trust her (Pulmonologist of IHN 1)*; but also on the patient's recognition of the received COPD care: *The patient was delighted and I am delighted with him; patients with COPD show recognition because they notice that we are constantly doing things for them (GP of IHN 2)*. A number of patients (especially of IHN 1 and IHN 3), however, highlighted that a bond with their assigned physician was missing; caused by the high turnover of personnel; but also by the insufficient receipt of information on their condition and limited regularity with their secondary care physicians. As patients, pulmonologists considered that infrequently programmed visits hindered the establishment of a relationship based on mutual trust with COPD patients. GPs confirmed that lack of time distracting from attending the patient properly: *The way the health care system of Catalonia works at the moment, it's impossible to provide quality care (…) I have arrived to see a hundred persons in two hours (GP of IHN 1)*.

### Interrelation of continuity of care types

**Figure 1.**
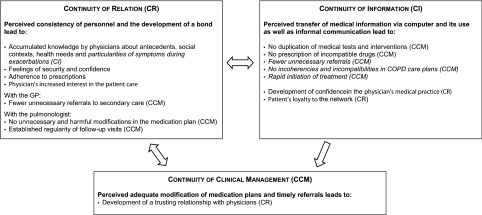
Consequences of the continuity of care types and its interrelation as perceived by COPD patients and their health professionals (*in italics*).

A series of consequences of continuity of care were identified by patients and physicians that were interlinked across its types (Figure 1), e.g., patients perceived that consistency of personnel with their GP or pulmonologist led to the accumulated knowledge of the patient's antecedents: *My GP, without having to look at the computer, knows what illness I suffer from (…) whereas, if the physicians are changed constantly, the physician doesn't know and you don't build trust (Patient of IHN 3)*; as well as avoided unnecessary secondary care referrals by the GP, who showed interest in the patient care and tried to solve the health problem himself/herself. In turn, correct modifications of medication plans and timely referrals favoured the development of a trusting relationship due to a perceived adequate medical practice: *My GP sent me to the specialists to whom he had to refer me, so, for me, he is a good doctor (Patient of IHN 1)*. The sharing and use of information was perceived by patients to avoid duplication of tests and the prescription of incompatible drugs. Physicians highlighted that information transfer avoided unnecessary referrals: *We usually don't refer patients back to primary care if there is nothing new. I write it down in the clinical history and say to the lady that she should see the GP on a regular basis. So the GP will open the clinical history and look it up. I got used to that because I don't like to refer the patient with a short note when the GP can read it anyway in the clinical history (Pulmonologist of IHN 1).*

## Discussion

This study presents first results of the performance of IHN regarding continuity of care considering its three types (continuity of clinical management, information and relation), which were analysed in four Catalan IHN with differences in the integration depth, inter-organisational relationship and implemented care coordination mechanisms, based on case studies consisting of COPD patients. By adopting a case study design, the phenomenon of continuity of care was understood in a multifaceted manner using different sets of information [45].

### Continuity of care is present in the studied health care networks

The analysis of the cases suggests that continuity of clinical management was existent in all networks due clear distribution of roles for COPD care across levels, rapid access during exacerbations and referrals to secondary care when needed; nevertheless, patients of some IHN highlighted too long waiting times to non-urgent secondary care. Concerning continuity of information, COPD patients of all networks perceived that their clinical information was transferred across levels via computer and that informal communication mechanisms were used (e-mail, telephone); however, also highlighted insufficient uptake of information by emergency physicians. Finally, regarding continuity of relation, only patients perceiving consistency of personnel, developed a close and trusting relationship with their professionals.

The results suggest a generally perceived adequate performance regarding continuity of clinical management and information in all of the studied integrated networks; showing similar results as in a Canadian study where patients reported less care coordination problems among providers and less information gaps in high versus low integrated networks [38] and as in a Catalonian study where a high number of patients reported that the GP was aware of instructions received from the secondary care level [29] and that care was coordinated when they were attended to in the organisation that managed both primary and secondary care services [34]. Even if results and literature might indicate better performance of IHN compared to other contexts, for example in England [52] and the United States [53] where patients highlighted significant discontinuity elements, attention should be drawn to the breakdowns identified by the informants of the present study and the Catalonian study [34] in terms of low accessibility to secondary care and the insufficient use of clinical information by physicians.

### Organisational and professionals related factors influence continuity of care

A series of factors were identified by the informants that influenced the perceived (non-)existence of continuity across care levels and can be attributed to the organisation and to the physicians. Regarding factors related to the organisation, informants highlighted that the inconsistency and shortage of personnel as well as time constraints influenced continuity of clinical management; and co-location of providers and implemented mechanisms, such as the electronic medical record system or electronic COPD patient registers, facilitated the sharing of information across levels. Concerning factors related to the physician, informants considered that mutual knowledge of physicians enhanced continuity of information, and that the lack of willingness to communicate and collaborate hindered both continuity of clinical management and continuity of information. Some of these factors have already been identified in different studies conducted in Northern Ontario [27] and British Columbia [54] in Canada and Belgium [28], such as co-location of providers [27,54], the shortage of health professionals or lack of time [27,28].

Regarding continuity of relation, especially factors related to the physicians favoured the development of a trusting relationship over time, as it was, the provided technical quality, the physician's positive attitude and the effective patient–physician communication, together with an established regularity of visits. Identified organisational factors related to the high turnover and inconsistency of personnel, which distracted from developing a relationship based on trust. Consistency of personnel is considered to be one of the two dimensions of continuity of relation [18], however, according to our results, might be rather seen as a relevant influencing factor of continuity of care in general. Results coincide with literature that a positive consultation experience (patients’ personal experience with physicians during consultations) is necessary to develop a relationship encompassing knowledge, trust, loyalty and regard; which can only be maintained when the patient is seen by the same physician over time (longitudinal care) [55].

### Clear distribution of responsibilities for COPD care but need for some improvements

COPD patients considered that responsibilities of care were clearly defined and distributed across levels so that both their GP and their pulmonologist assume differing, not overlapping tasks in managing their condition. Yet, the pulmonologist was identified to be mainly responsible for the overall COPD care. Similarly, in a patient survey conducted in the same region, 41% of interviewees identified the secondary care physician and 45% both primary and secondary care physicians as the main responsible professional(s) for providing care [29]. Patients’ mentioned rationales for a high responsibility assigned to pulmonologists related to their expert knowledge and competence, as confirmed elsewhere [24]. A further explanation could be that the assignation of COPD care responsibility is dependent on the developed patient–physician relationship [24] and frequency of contact [26]. Nevertheless, in practice, some discrepancies regarding the delivery of acute care and follow-up after hospitalisation were identified that could indicate pitfalls in the use of mechanisms put in place, e.g., for the standardisation of work processes (clinical COPD guidelines and care pathways) [11] and should therefore be addressed to improve the coordination of COPD care.

### Drawing a global picture of inter-related consequences

Although understanding the complexity of continuity of care is crucial to improving it in practice [19], the interrelation of continuity types has scarcely been studied. Previous studies primarily analysed trade-off preferences in terms of balancing seeing a preferred provider with fast access to health care services [56–58]. This study contributed to the understanding of the inter-dependency of all three continuity types, building upon a first attempt developed in a meta-synthesis [22]. Results suggest that continuity of relation played an important role by influencing continuity of clinical management and information - as identified elsewhere [18,24,59], such as better access or proactive COPD follow-up [24] - but also vice versa: the perception of the uptake of information from electronic medical records and adequate and timely referrals favoured the development of a close and trusting patient–physician relationship. Although continuity of relation can difficultly be analysed across care level (being rather relevant with one or more physician(s) of one care level), this continuity type should be paid special attention since it seems to facilitate continuity of clinical management and information, also when different professionals are involved in the provision of care.

### Patients’ capacity of evaluating the health services

Triangulation of patient's perceptions with those of health professionals’ and reviews of medical records generally shows congruency of findings; thus supporting the notion of the health care users’ ability to evaluate services [38,60]; however triangulation also presents a few conflicting results, especially with respect to the distribution of responsibilities and information transfer and use across levels. Possible explanations might be that some aspects of continuity are less salient to health care users [61,62], conflicts or perceived problems among professionals do not have an impact on the care itself or patients simply present different health needs regarding continuity, versus coordination of care.

### Study limitations

Two study limitations should be noted. First, data were provided by a cluster of patients with relatively similar characteristics (elderly male patients) due to the limited number of female patients that responded to selection criteria and accepted to participate; which might have restricted the discursive variation and excluded any additional elements explaining the perception of continuity of care. Nevertheless, existent results are not conclusive if the perception of continuity is dependent on age, sex or socio-economic status [63]. We suggest investigating perceptions of female and younger patients with different conditions and considering different socio-economic levels in order to expand the discourse about continuity of care in health care networks.

As a second study limitation, it should be noted that the performance of IHN were only analysed by comparing different types. Nevertheless, a comparison of IHN with other organisations of the national health system would help to better understand the phenomenon of continuity of care in different contexts. Further research could provide a clearer picture, not only about the functioning of different types of networks but also their potential benefits over less integrated systems.

### Recommendations for the health care organisations

In this study, a series of factors linked with the organisation and the professionals were identified. Addressing these factors should initially improve the coordination of services across care levels and subsequently the patient experiences of a smooth trajectory, for example by improving working conditions, providing physicians with additional time to use the coordination mechanisms or encouraging contact between professionals to enhance the development of relationships, which should stimulate both formal and informal communication and interdisciplinary collaboration in care management processes; as it was postulated by different authors [40,64–66].

## Conclusion

This article contributes to the existent knowledge of IHN performance by analysing continuity of care based on case studies of COPD patients. Findings suggest the presence of continuity of care but also the existence of a series of noteworthy difficulties related to all types of continuity. The different identified factors attributed to the organisation and the physicians shed light on where to direct efforts to enhance the coordination of services, thus study findings can provide opportunities to improving the care process of COPD patients specifically, but also the care process of patients with other conditions who receive both primary and secondary care. Given the scarcity of evidence-based information on the performance of IHN continuity of care should be subject to further research to advance in its knowledge and to develop and implement strategies for more patient-centred integrated health care systems.

## Figures and Tables

**Table 1.**
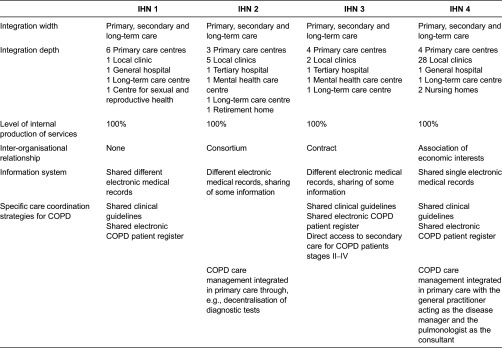
Key characteristics of selected IHN

COPD, chronic obstructive pulmonary disease; IHN, integrated health care network.

Source: Vázquez et al. [10,11].

**Table 2.**
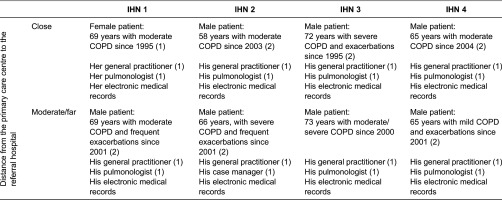
Description of the cases and their source of information in each integrated health care network

COPD, chronic obstructive pulmonary disease; IHN, integrated health care network; (…) Number of interviews per informant.
